# Genetic Insights Into Allergic Contact Dermatitis: Reassessing the Role of LCE3C_LCE3B Deletion

**DOI:** 10.1111/cod.70058

**Published:** 2025-12-14

**Authors:** Zeineb Ben Lamine, Amen Moussa, Marwa Bouhoula, Razene Gerisha, Sarra Sabbagh, Imen Kacem, Asma Aloui, Souheil Chatti, Aicha Brahem, Ramzi Zemni, Foued Ben Hadj Slama

**Affiliations:** ^1^ Immunogenetics Unit, Faculty of Medicine Ibn Al Jazzar University of Sousse Sousse Tunisia; ^2^ Regional Blood Transfusion Center of Sousse University of Sousse Sousse Tunisia; ^3^ Occupational Medicine Department, Faculty of Medicine of Monastir University of Monastir Sousse Tunisia; ^4^ Occupational Medicine Department University Hospital of Farhat Hached University of Sousse Sousse Tunisia; ^5^ Genetics Laboratory, Sahloul Hospital University of Sousse Sousse Tunisia

**Keywords:** allergic contact dermatitis, genetic susceptibility, LCE3C_LCE3B deletion, polysensitisation

## Abstract

**Background:**

Allergic contact dermatitis (ACD) is a multifactorial inflammatory skin disorder. Polysensitisation, defined as hypersensitivity to ≥ 3 unrelated allergens, reflects a more severe clinical form. The LCE3C_LCE3B deletion, implicated in skin barrier dysfunction, has a yet unclear role in the susceptibility to ACD and polysensitisation.

**Objective:**

This study aims to evaluate the association between LCE3C_LCE3B deletion and susceptibility to ACD and polysensitisation in the Tunisian population.

**Methods:**

A case–control study included 94 confirmed ACD patients and 125 age‐ and sex‐matched controls without immune‐related diseases. Patch testing followed the European Baseline Series. LCE3C_LCE3B genotyping was performed using conventional three‐primer PCR after DNA extraction by the salting‐out method.

**Results:**

Polysensitisation was observed in 33% of ACD cases. Genotyping of the LCE3C_LCE3B deletion did not reveal statistically significant differences in allelic or genotypic frequencies between ACD cases and controls. Furthermore, no statistically significant association was found between the LCE3C_LCE3B del and polysensitisation. Similarly, no significant associations were observed between the LCE3C_LCE3B deletion and sensitisation to metals, including nickel, cobalt and chromium.

**Conclusion:**

No significant association was found between the LCE3C_LCE3B deletion and either ACD or polysensitisation. Larger studies are needed to clarify the genetic contribution to these conditions.

## Graphical abstract

This study found no significant association between the LCE3C_LCE3B deletion and allergic contact dermatitis or polysensitisation in Tunisian patients, suggesting that other genetic or environmental factors may contribute to disease susceptibility.

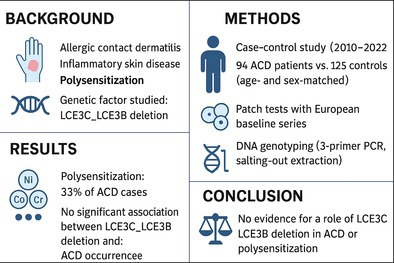

## 1 Introduction

Allergic contact dermatitis (ACD) is a common inflammatory skin disorder characterised by a delayed‐type (type IV) hypersensitivity reaction to low‐molecular‐weight allergens. Epidemiological studies estimate that ACD affects approximately 20% of the European population [1], with a prevalence ranging from 9.2% to 35.2% in North America and reaching 20.6% in China [2, 3, 4]. In Tunisia, Belhadj et al. reported a 55.7% positivity rate to contact allergens among patients, suggesting a high regional disease burden [5]. ACD is increasingly recognised not only for its dermatological impact but also for its significant consequences on quality of life, work productivity and mental health [6]. It is a multifactorial condition resulting from the complex interaction between genetic predisposition and environmental factors [7, 8, 9]. The pathophysiology of ACD is influenced by the skin's ability to maintain an effective barrier against external agents. This barrier function is primarily regulated by genes within the epidermal differentiation complex (EDC), located on chromosome 1q21 [10, 11, 12]. Structural proteins, such as filaggrin and the Late Cornified Envelope (LCE) family members, are important for maintaining skin integrity and preventing allergen penetration. In particular, deletions within the LCE gene cluster, especially the LCE3C_LCE3B deletion (LCE3C_LCE3B‐del), have been associated with skin barrier dysfunction and an increased susceptibility to inflammatory skin conditions, including psoriasis and atopic dermatitis [13, 14]. However, the role of LCE3C_LCE3B deletions in ACD and, more specifically, in polysensitisation, a clinically more severe phenotype characterised by hypersensitivity to three or more chemically unrelated allergens, remains insufficiently explored [15, 16].

This study aims to evaluate the association between the LCE3C_LCE3B deletion and susceptibility to polysensitisation. To our knowledge, this is the first study to address this question in the North African population, providing insights that could inform both genetic counselling and occupational health interventions.

## 2 Methods

### 2.1 Study Design and Patient Recruitment

A case–control study was conducted from September 2022 to December 2023 at the Occupational Medicine Department of Farhat Hached University Hospital (Sousse, Tunisia). It included 94 patients with confirmed ACD and 125 age‐ and sex‐matched healthy controls recruited from voluntary blood donors at the regional blood bank. Controls were free of any personal or family history of allergic, dermatological or autoimmune diseases. All participants were of North African ancestry.

### 2.2 Patch Testing

Patch testing was performed using the European Baseline Series (ECB‐2000), applied to the upper back for 48 h under occlusion. Readings were taken at D2 and D3 and interpreted using the International Contact Dermatitis Research Group (ICDRG) criteria. Sensitisation was defined as a positive reaction to ≥ 1 allergen. Patients were classified as monosensitised, bisensitised, or polysensitised based on reactivity to unrelated allergens [17, 18].

### 2.3 Genotyping

Genomic DNA was extracted using the salting‐out method. LCE3C_LCE3B deletion genotyping was performed using a conventional three‐primer PCR, with primers from de Cid et al. [19]. The sequences of the primers used are listed in Table 1. The PCR protocol for LCE3C_LCE3B deletion genotyping started with an initial denaturation at 94°C for 5 min, followed by 34 cycles of 94°C for 30 s, 54°C for 30 s, and 72°C for 30 s, with a final extension at 72°C for 7 min. The undeleted allele yielded a 240 bp product, and the deleted allele yielded 199 bp.

**TABLE 1:** Primers sequences [19].

Primers	Primer sequences
LCE3CF	5′ TCACCCTGGAACTAGACCTCA 3′
LCE3CR	5′ CTCCAACCACTTGTTCTTCTCA 3′
LCE3CR2D	5′ CATCCCAGGGATGCTGCATG 3′

### 2.4 Statistical Analysis and Ethical Considerations

Data were analysed using SPSS v28. Statistical analyses, including Chi‐square tests and logistic regression, were performed to identify associations, with a significance threshold of *p* < 0.05. The study protocol was approved by the local ethics committee (Committee for Ethics in Medical and Scientific Research, CEFMS 234/2024) and was conducted in accordance with the Declaration of Helsinki. All participants (patients and controls) provided written informed consent, and confidentiality and anonymity were strictly maintained throughout the study.

## 3 Results

### 3.1 Demographic Data and Sensitisation Profile

The mean age of ACD patients was 42.06 ± 12.58 years, with a female predominance (63.8%). Patch testing revealed potassium dichromate (*n* = 35; 37.2%), nickel sulfate (*n* = 33; 35.1%) and cobalt chloride (*n* = 19; 20.2%) as the most common allergens. Among ACD patients, 33% were polysensitised, while 39.4% and 27.7% were mono‐ and bisensitised, respectively. The most frequent allergens identified were metals (64.9%), preservatives (20.2%), fragrances (30.9%) and rubber additives. The detailed distribution of patch test results is shown in Tables 2 and 3.

**TABLE 2:** Distribution of cases according to patch test results.

Patch test results	*N* (%)
Allergens	
Potassium dichromate 0.5% pet	35 (37.2)
Nickel sulfate hexahydrate 5.0% pet	33 (35.1)
Cobalt chloride hexahydrate 1.0% pet	19 (20.2)
Peru Balsam 25.0% pet	12 (12.7)
Benzisothizolinone 0.1% pet	9 (9.5)
Methyldibromo glutaronitrile 0.5% pet	8 (8.5)
Thiuram mix 1.0% pet	8 (8.5)
4‐tert‐butylphenol‐formaldehyde resin (PTBP) 1.0% pet	8 (8.5)
Colophanium 20.0% pet	7 (7.5)
Formaldehyde 2.0% aq	7 (7.5)
Fragrance Mix 8.0% pet	7 (7.5)
Mercaptobenzothiazole (MBT) 2.0% pet	7 (7.5)
Sodium metabisulfite 1.0% pet	6 (6.4)
Textile dye mix 6.6% pet	6 (6.4)
Mercapto mix 2.0% pet	5 (5.3)
Sesquiterpene lactone mix 0.1% pet	5 (5.3)
Laury polyglucose 3.0% pet	4 (4.2)
2‐n‐Octyl‐4‐isotiazolin‐3‐one 0.1% pet	4 (4.3)
Composite mix II 2.5% pet	3 (3.2)
Decyl glucoside 5.0% pet	3 (3.2)
Fragrance mix II 14.0% pet	3 (3.2)
Linalool hydroperoxides 1.0% pet	3 (3.3)
Methylisothiazolinone + methylchloroisothiazolinone 0.02% aq	3 (3.2)
N‐Isoprpyl‐N‐phenyl‐4‐phenylenediamine (IPPD) 0.1% pet	3 (3.2)
2 Hydroxyethyl methacrylate 2.0% pet	3 (3.2)
Caine mix III 10.0% pet	2 (2.1)
Epoxy resin, Bisphenol A1 1.0% pet	2 (2.2)
Lanolin Alcohol 30.0% pet	2 (2.2)
Methylisothiazolinone 0.2% aq	2 (2.2)
Paraben mix 16.0% pet	2 (2.1)
Paraphenylenediamine (PPD) 1.0% pet	2 (2.1)
Budesonide 0.01% pet	1 (1.1)
Linalool hydroperoxides 0.5% pet	1 (1.1)
Limonene hydroperoxides 0.3% pet	1 (1.1)
Limonene hydroperoxides 0.2% pet	1 (1.1)
Neomycin sulfate 20.0% pet	1 (1.1)
Propolis 10.0% pet	1 (1.1)
Quaternium 15 1.0% pet	1 (1.1)
Allergens subsets	
Metals	61 (64.9)
Fragrance compounds	29 (30.9)
Preservatives	19 (20.2)
Vulcanization accelerators	16 (17)
Synthetic polymers	13 (13.8)
Colorants	8 (8.5)
Pharmaceuticals	4 (4.3)
Sensitization status	
Poly‐sensitization	31 (33)
Bisensitized	26 (27.7)
Monosensitized	37 (39.4)

Abbreviations: Aq, aqueous solution; Pet, petrolatum.

**TABLE 3:** Sensitisation patterns by allergens.

Allergens	Non polysensitized *N* (%)	Polysensitized *N* (%)
Potassium dichromate 0.5% pet	16 (45.7)	19 (54.3)
Nickel sulfate hexahydrate 5.0% pet	15 (45.5)	18 (54.5)
Cobalt chloride hexahydrate 1.0% pet	7 (36.8)	12 (63.2)
Peru Balsam 25.0% pet	4 (33.3)	8 (66.7)
Benzisothizolinone 0.1% pet	7 (77.8)	2 (22.2)
Methyldibromo glutaronitrile 0.5% pet	4 (50)	4 (50)
Thiuram mix 1.0% pet	2 (25)	6 (75)
4‐tert‐butylphenol‐formaldehyde resin (PTBP) 1.0% pet	3 (37.5)	5 (62.5)
Colophanium 20.0% pet	3 (42.9)	4 (57.1)
Formaldehyde 2.0% aq	2 (28.6)	5 (71.4)
Fragrance mix 8.0% pet	1 (14.3)	6 (85.7)
Mercaptobenzothiazole (MBT) 2.0% pet	0 (0)	7 (100)
Sodium metabisulfite 1.0% pet	4 (66.7)	2 (33.3)
Textile dye mix 6.6% pet	4 (66.7)	2 (33.3)
Mercapto mix 2.0% pet	0 (0)	5 (100)
Sesquiterpene lactone mix 0.1% pet	1 (20)	4 (80)
Laury Polyglucose 3.0% pet	3 (75)	1 (25)
2‐n‐Octyl‐4‐isotiazolin‐3‐one 0.1% pet	1 (25)	3 (75)
Composite mix II 2.5% pet	1 (33.3)	2 (66.7)
Decyl glucoside 5.0% pet	1 (33.3)	2 (66.7)
Fragrance mix II 14.0% pet	0 (0)	3 (100)
Linalool hydroperoxides 1.0% pet	0 (0)	3 (100)
Methylisothiazolinone + methylchloroisothiazolinone 0.02% aq	1 (33.3)	2 (66.7)
N‐Isoprpyl‐N‐phenyl‐4‐phenylenediamine (IPPD) 0.1% pet	3 (100)	0 (0)
2 Hydroxyethyl methacrylate 2.0% pet	1 (33.3)	2 (66.7)
Caine mix III 10.0% pet	2 (100)	0 (0)
Epoxy resin, Bisphenol A1 1.0% pet	0 (0)	2 (100)
Lanolin alcohol 30.0% pet	0 (0)	2 (100)
Methylisothiazolinone 0.2% aq	1 (50)	1 (50)
Paraben mix 16.0% pet	0 (0)	2 (100)
Paraphenylenediamine (PPD) 1.0% pet	1 (50)	1 (50)
Budesonide 0.01% pet	1 (100)	0 (0)
Linalool hydroperoxides 0.5% pet	0 (0)	1 (100)
Limonene hydroperoxides 0.3% pet	0 (0)	1 (100)
Limonene hydroperoxides 0.2% pet	0 (0)	1 (100)
Neomycin sulfate 20.0% pet	0 (0)	1 (100)
Propolis 10.0% pet	0 (0)	1 (100)
Quaternium 15 1.0% pet	0 (0)	1 (100)

Aq, aqueous solution; Pet, petrolatum.

### 3.2 Genetic Analysis: Allele and Genotype Distribution

Allele frequencies in both the patient and control groups conformed to Hardy–Weinberg equilibrium. The deletion allele (D^+^) was present in 55.85% of ACD cases and 58.87% of controls. No statistically significant differences were observed in allelic or genotypic distributions between ACD patients and controls across any genetic model (codominant, dominant or recessive) (Table 4). Similarly, no significant associations were found between the deletion and polysensitisation status among ACD patients (Table 5).

**TABLE 4:** Association of deletion of LCE3C‐3B genes with ACD.

	Cases (*n*, %)	Controls (*n*, %)	OR (95% CI)	*p*
Allele				
D−	83 (44.15%)	102 (41.13%)	1	0.52
D+	105 (55.85%)	146 (58.87%)	0.88 [0.6–1.29]	
Genotype				
D−D−	20 (21.28%)	25 (20%)	1	0.77
D+D−	43 (45.74%)	52 (42.4%)	1.03 [0.51–2.10]	
D+D+	31 (32.98%)	47 (37.6%)	0.82 [0.39–1.72]	
Dominant model				
D−D−	20 (21.28%)	25 (20%)	1	0.84
D+D− and D+D+	74 (78.72%)	99 (80%)	0.93 [0.48–1.8]	
Recessive model				
D+D− and D−D−	63 (67.02%)	77 (62.4%)	1	0.45
D+D+	31 (32.98%)	47 (37.6%)	0.8 [0.46–1.41]	

**TABLE 5:** Association of deletion of LCE3C‐3B genes with polysensitisation.

	Polysensitised (*n*, %)	Controls (*n*, %)	OR (95% CI)	*p*
Allele				
D−	25 (40.32%)	58 (46.03%)	1	0.46
D+	37 (59.68%)	68 (53.97%)	1.26 [0.68–2.34]	
Genotype				
D−D−	5 (16.13%)	15 (23.8%)	1	0.72
D+D−	15 (48.39%)	28 (44.4%)	1.61 [0.49–5.29]	
D+D+	11 (35.48%)	20 (31.8%)	1.65 [0.47–5.77]	
Dominant model				
D−D−	5 (16.13%)	15 (23.8%)	1	0.39
D+D− and D+D+	26 (83.87%)	48 (76.2%)	1.62 [0.53–4.97]	
Recessive model				
D+D− and D−D−	20 (64.52%)	43 (68.2%)	1	0.72
D+D+	11 (35.48%)	20 (31.8%)	0.84 [0.34–2.09]	

No significant association was found between the LCE3C_LCE3B deletion and sensitisation profiles, including polysensitisation and mono/bi‐sensitisation. Specifically, no significant association was observed with sensitisation to nickel, chromium or cobalt, with *p*‐values consistently greater than 0.05 across all genetic models (allelic, genotypic, dominant and recessive) (Tables 6, 7, 8, 9).

**TABLE 6:** Association of deletion of LCE3C‐3B genes with metal sensitisation.

	Metal sensitisation (*n*, %)	Controls (*n*, %)	OR (95% CI)	*p*
Allele				
D−	53 (43.44%)	103 (41.2%)	1	0.68
D+	69 (56.56%)	147 (58.8%)	0.91 [0.59–1.4]	
Genotype				
D−D−	10 (16.39%)	25 (20%)	1	0.32
D+D−	33 (54.1%)	53 (42.4%)	1.56 [0.66–3.65]	
D+D+	18 (29.51%)	47 (37.6%)	0.96 [0.38–2.38]	
Dominant model				
D−D−	10 (16.39%)	25 (20%)	1	0.55
D+D− and D+D+	51 (83.61%)	100 (80%)	1.27 [0.57–2.86]	
Recessive model				
D+D− and D−D−	43 (70.49%)	78 (62.4%)	1	0.27
D+D+	18 (29.51%)	47 (37.6%)	0.69 [0.36–1.34]	

**TABLE 7:** Association of deletion of LCE3C‐3B genes with nickel sensitisation.

	Nickel sensitisation (*n*, %)	Controls (*n*, %)	OR (95% CI)	*p*
Allele				
D−	29 (43.94%)	103 (41.2%)	1	0.69
D+	37 (56.06%)	147 (58.8%)	0.89 [0.52–1.54]	
Genotype				
D−D−	5 (15.15%)	25 (20%)	1	0.3
D+D−	19 (57.58%)	53 (42.4%)	1.79 [0.60–5.35]	
D+D+	9 (27.27%)	47 (37.6%)	0.96 [0.29–3.17]	
Dominant model				
D−D−	5 (15.15%)	25 (20%)	1	0.53
D+D− and D+D+	28 (84.85%)	100 (80%)	1.4 [0.49–3.99]	
Recessive model				
D+D− and D−D−	24 (72.73%)	78 (62.4%)	1	0.27
D+D+	9 (27.27%)	47 (37.6%)	0.62 [0.27–1.45]	

**TABLE 8:** Association of deletion of LCE3C‐3B genes with chromium sensitisation.

	Chromium sensitisation (*n*, %)	Controls (*n*, %)	OR (95% CI)	*p*
Allele				
D−	33 (48.53%)	103 (41.2%)	1	0.28
D+	35 (51.47%)	147 (58.8%)	0.74 [0.43–1.27]	
Genotype				
D−D−	8 (23.53%)	25 (20%)	1	0.48
D+D−	17 (50%)	53 (42.4%)	1 [0.38–2.63]	
D+D+	9 (26.47%)	47 (37.6%)	0.6 [0.21–1.74]	
Dominant model				
D−D−	8 (23.53%)	25 (20%)	1	0.65
D+D− and D+D+	26 (76.47%)	100 (80%)	0.81 [0.33–2]	
Recessive model				
D+D− and D−D−	25 (73.53%)	78 (62.4%)	1	0.23
D+D+	9 (26.47%)	47 (37.6%)	0.6 [0.26–1.39]	

**TABLE 9:** Association of deletion of LCE3C‐3B genes with cobalt sensitisation.

	Cobalt sensitisation (*n*, %)	Controls (*n*, %)	OR (95% CI)	*p*
Allele				
D−	14 (36.84%)	103 (41.2%)	1	0.61
D+	24 (63.16%)	147 (58.8%)	1.2 [0.59–2.43]	
Genotype				
D−D−	2 (10.53%)	25 (20%)	1	0.55
D+D−	10 (52.63%)	53 (42.4%)	2.36 [0.48–11.58]	
D+D+	7 (36.84%)	47 (37.6%)	1.86 [0.36–9.64]	
Dominant model				
D−D−	2 (10.53%)	25 (20%)	1	0.53
D+D− and D+D+	17 (89.47%)	100 (80%)	2.13 [0.45–9.8]	
Recessive model				
D+D− and D−D−	12 (63.16%)	78 (62.4%)	1	0.94
D+D+	7 (36.84%)	47 (37.6%)	0.96 [0.36–2.6]	

## 4 Discussion

Despite uniform allergen exposure, only some individuals develop ACD, indicating a role for genetic predisposition. Polymorphisms in genes related to immune regulation, xenobiotic metabolism, and skin barrier integrity have been extensively studied in ACD [20]. Polysensitisation, defined as sensitisation to multiple unrelated allergens, represents a clinically significant phenotype in ACD, often associated with increased disease severity and broader exposure risks [21, 22]. Polysensitisation, as a more severe and generalised form of sensitisation, may represent a genetically distinct subgroup with heightened susceptibility to allergen‐induced immune activation [8]. Indeed, experimental models show that polysensitised individuals exhibit lower elicitation thresholds and greater sensitivity to new allergens like dinitrochlorobenzene (DNCB) compared to monosensitised individuals [8]. Genetic variants in pro‐inflammatory cytokine genes, such as TNFA (rs1800629) and IL16 (rs4778889), have been associated with increased risk of para‐compound sensitisation and polysensitisation [23]. In this study, we extended this inquiry by examining variants affecting skin barrier function, particularly the LCE3C‐LCE3B deletion, a structural variant previously implicated in psoriasis and atopic dermatitis [11, 24]. The LCE gene cluster on chromosome 1q21.3 encodes late cornified envelope proteins essential for stratum corneum formation and epidermal barrier integrity [25]. Within this cluster, LCE3C and LCE3B play critical roles in maintaining mechanical resilience and antimicrobial defense [26]. A 32‐kb deletion encompassing these genes (LCE3C_LCE3B‐del) has been shown to compromise skin barrier function, potentially facilitating allergen penetration and immune activation [11].

To our knowledge, this study is the first to investigate the association between LCE3C_LCE3B‐del and ACD and polysensitisation in a North African population. Although our study did not find a statistically significant association between the LCE3C_LCE3B deletion and ACD or polysensitisation, this negative result is still informative. It suggests that, in this North African cohort, the deletion does not appear to be a major risk factor. Previous work by Molin et al. reported a higher prevalence of this deletion in ACD patients compared to controls (51.4% vs. 32.8%) [12], highlighting the importance of validating these findings in different populations. Interestingly, the deletion frequency in our Tunisian cohort (56%) aligns more closely with Caucasian populations (59% in France) than with sub‐Saharan African groups (28%–35%) [27], indicating possible admixture or distinct evolutionary pressures in North Africa. This finding underscores the importance of considering population‐specific genetic backgrounds in studies of skin barrier‐related diseases. We also explored whether LCE3C_LCE3B‐del correlated with sensitisation to specific metals (nickel, cobalt, chromium), but no significant associations emerged. Potential explanations include compensatory upregulation of other LCE genes, such as LCE3A, which has been shown to increase expression in the presence of the deletion and possesses antimicrobial properties that may mitigate barrier dysfunction [28]. Additionally, other barrier‐related genes, such as FLG (encoding filaggrin), or tight junction components like claudin‐1, may play more prominent roles in ACD susceptibility in our population [13].

Nickel sensitisation has been associated with both immune‐related and skin‐barrier genes. A genome‐wide association study identified PELI1 (rs6733160, OR = 6.44) and NTN4 (rs2367563, OR = 0.17) as significant loci [29]. Variants in CLDN1 (rs9290927) were linked to nickel sensitisation in individuals without ear piercings (*p* = 0,013) [30]. Filaggrin loss‐of‐function mutations (R501X, 2282del4) showed heterogeneous results: In a population‐based study, people with FLG mutations were more likely to be sensitised to nickel, especially if they also reported intolerance to fashion jewellery [31]. Patients with atopic dermatitis (AD) carrying these mutations exhibited a modest overall risk (OR 1.65, 95% CI 0.88–3.09), which became significant in AD patients without ear piercings (*χ* ^2^ = 12.14), highlighting the role of skin barrier impairment in nickel sensitisation [32]. In fact, lack of filaggrin reduces the skin's ability to bind nickel and maintain an effective barrier, which could make it easier for nickel to penetrate and worsen hand eczema in atopic dermatitis patients [32]. However, in a dermatology clinic, no clear link was found between FLG mutations and nickel allergy, polysensitisation, or allergic contact dermatitis, although carriers tended to develop disease earlier and for longer [33]. Chromium sensitisation, has been related to pro‐inflammatory and detoxification pathways: TNFA promoter polymorphism (−308 G/A, OR 3.9) and GSTT1 null genotype (OR 5.5) were significantly associated, while GSTM1 yielded variable results [34].

Some limitations must be acknowledged. The modest sample size limits statistical power. Functional studies investigating how LCE3C_LCE3B‐del affects allergen penetration, immune activation, and microbial composition could provide mechanistic insights. Furthermore, genome‐wide association studies integrating skin barrier, immune and metabolic pathways may identify novel loci contributing to polysensitisation.

## 5 Conclusion

This study did not identify any significant association between the LCE3C_LCE3B deletion and the occurrence of ACD or polysensitisation in the Tunisian population. Although our findings do not support a role for this genetic variant in ACD susceptibility, further studies involving larger and more diverse cohorts are necessary to explore other potential genetic contributors to polysensitisation and allergic contact dermatitis.

## Funding

The authors received no specific funding for this work.

## Conflicts of Interest

The authors declare no conflicts of interest.

## Data Availability

The data that support the findings of this study are available from the corresponding author upon reasonable request.
